# Pim-1 Kinase Phosphorylates and Stabilizes 130 kDa FLT3 and Promotes Aberrant STAT5 Signaling in Acute Myeloid Leukemia with FLT3 Internal Tandem Duplication

**DOI:** 10.1371/journal.pone.0074653

**Published:** 2013-09-05

**Authors:** Karthika Natarajan, Yingqiu Xie, Mehmet Burcu, Douglas E. Linn, Yun Qiu, Maria R. Baer

**Affiliations:** 1 Greenebaum Cancer Center, University of Maryland, Baltimore, Maryland, United States of America; 2 Department of Pharmacology and Experimental Therapeutics, University of Maryland School of Medicine, Baltimore, Maryland, United States of America; 3 Department of Medicine, University of Maryland School of Medicine, Baltimore, Maryland, United States of America; Emory University, United States of America

## Abstract

The type III receptor tyrosine kinase fms-like tyrosine kinase 3 (FLT3) is expressed on both normal hematopoietic stem cells and acute myeloid leukemia (AML) cells and regulates their proliferation. Internal tandem duplication (ITD) mutation of FLT3 is present in a third of AML cases, results in constitutive activation and aberrant signaling of FLT3, and is associated with adverse treatment outcomes. While wild-type (WT) FLT3 is predominantly a 150 kDa complex glycosylated cell surface protein, FLT3-ITD is partially retained in the endoplasmic reticulum as a 130 kDa underglycosylated species associated with the chaperones calnexin and heat shock protein (HSP) 90, and mediates aberrant STAT5 signaling, which upregulates the oncogenic serine/threonine kinase Pim-1. FLT3 contains a Pim-1 substrate consensus serine phosphorylation site, and we hypothesized that it might be a Pim-1 substrate. Pim-1 was indeed found to directly interact with and serine-phosphorylate FLT3. Pim-1 inhibition decreased the expression and half-life of 130 kDa FLT3, with partial abrogation by proteasome inhibition, in association with decreased FLT3 binding to calnexin and HSP90, and increased 150 kDa FLT3 expression and half-life, with abrogation by inhibition of glycosylation. These findings were consistent with Pim-1 stabilizing FLT3-ITD as a 130 kDa species associated with calnexin and HSP90 and inhibiting its glycosylation to form the 150 kDa species. Pim-1 knockdown effects were similar. Pim-1 inhibition also decreased phosphorylation of FLT3 at tyrosine 591 and of STAT5, and expression of Pim-1 itself, consistent with inhibition of the FLT3-ITD-STAT5 signaling pathway. Finally, Pim-1 inhibition synergized with FLT3 inhibition in inducing apoptosis of FLT3-ITD cells. This is, to our knowledge, the first demonstration of a role of Pim-1 in a positive feedback loop promoting aberrant signaling in malignant cells.

## Introduction

The type III receptor tyrosine kinase fms-like tyrosine kinase 3 (FLT3) is expressed on both normal hematopoietic cells and acute myeloid leukemia (AML) cells and regulates their proliferation [1]. FLT3 is mutated in leukemia cells of approximately a third of AML patients, most commonly by internal tandem duplication (ITD) within the juxtamembrane domain, resulting in constitutive activation and aberrant signaling [1], [2]. Patients with AML with FLT3-ITD have adverse treatment outcomes, and specifically short disease-free survival [2]. FLT3 inhibitors have activity in AML with FLT3-ITD [3], but the single randomized clinical trial reported to date did not demonstrate improved treatment outcome [4]. FLT3 inhibitors initially tested, including lestaurtinib [4], midostaurin [5] and sorafenib [6], are multikinase inhibitors, and the more selective FLT3 inhibitor AC220 [7] is currently undergoing initial clinical testing.

While wild-type FLT3 (FLT3-WT) is synthesized as a 130 kDa underglycosylated, or high-mannose, species, and is then folded in the endoplasmic reticulum (ER) and exported to the Golgi apparatus, where it is glycosylated to form a 150 kDa complex glycosylated species prior to translocation to the cell surface [8], [9], FLT3-ITD is partially retained in the ER as the underglycosylated 130 kDa species in association with the transmembrane ER chaperone calnexin [8]. FLT3-ITD also associates with the cytosolic chaperone heat shock protein (HSP) 90 [8], [10], [11], which protects it from ubiquitination and proteasomal degradation [12]. Thus in cells with FLT3-ITD, underglycosylated, or high-mannose, 130 kDa FLT3, localized intracellularly, is overexpressed in relation to complex glycosylated 150 kDa FLT3. The mislocalized, constitutively phosphorylated FLT3-ITD activates the signal transducer and activation of transcription (STAT) 5 signaling pathway [9], [13]. STAT5 signaling in turn causes transcriptional activation of its downstream target Pim-1 [14]–[16], a serine/threonine kinase encoded by a proto-oncogene originally identified as the proviral insertion site in Moloney murine leukemia virus lymphomagenesis [17]. Pim-1 protein is expressed as two isoforms with alternative translation initiation sites and molecular weights of 33 kDa and 44 kDa, though the 33 kDa species may predominate in human cells [18]. Pim-1 is synthesized in an active form by virtue of its hinge structure [19] so that its activity is regulated solely by its level of expression. Pim-1 is a member of the pim kinase family, which also includes Pim-2 and Pim-3 [20].

Pim-1 phosphorylates and thereby regulates a number of proteins that are important in key cellular processes, including the pro-apoptotic protein BAD [15], [21], the cell cycle regulatory proteins p21 [22], p27 [23], Cdc25A [24] and Cdc25C [25], the transcription factors SOCS-1 [26], RUNX3 [27] and c-myc [28], the chemokine receptor CXCR4 [29], and, as we previously demonstrated, the multidrug resistance-associated ATP-binding cassette (ABC) proteins ABCB1 or P-glycoprotein (Pgp) [30] and ABCG2 or breast cancer resistance protein (BCRP) [31]. Pim-1 phosphorylation of its substrate proteins p21 [22], SOCS-1 [26], RUNX3 [27] and c-myc [28] enhances their stability, and Pim-1 phosphorylation of ABCB1 and ABCG2 enhances the stability of intracellular ABCB1 and ABCG2 and promotes their cell surface translocation [30], [31]. Pim-1 phosphorylation also promotes cell surface translocation of CXCR4 [29].

FLT3 contains a putative Pim-1 substrate consensus phosphorylation site [32], [33] at serine 935 (RKRPS)**,** and this prompted us to test whether Pim-1, expressed downstream of FLT3-ITD [14]-[16], might regulate FLT3-ITD stability and expression in a positive feedback loop. We demonstrate here that Pim-1 directly interacts with and serine-phosphorylates FLT3-ITD and stabilizes it in its 130 kDa form, thereby promoting STAT5 activation and aberrant signaling in FLT3-ITD cells. Thus FLT3-ITD signaling occurs not only through Pim-1 upregulation and subsequent phosphorylation of its target proteins, but also through Pim-1-mediated serine phosphorylation and consequent stabilization and ER retention of 130 kDa FLT3-ITD. This is, to our knowledge, the first demonstration of a role of Pim-1 in a positive feedback loop promoting aberrant signaling in malignant cells. Moreover, our data provide additional support for the therapeutic potential of targeting Pim-1 kinase in AML with FLT3-ITD.

## Materials and Methods

### Cell lines

FLT3-ITD cell lines included MV4-11 and MOLM-14 [34], obtained from the American Type Culture Collection (ATCC), Manassas, VA, and FLT3-ITD-transfected Ba/F3 cells, or Ba/F3**-**ITD [35], cells obtained from Dr. Mark Levis, Johns Hopkins University School of Medicine, Baltimore, MD. Pim-1-expressing FLT3-WT EOL-1 cells [14] were also obtained from Dr. Levis. The PT67 cell line was purchased from Clontech Laboratories, Mountain View, CA.

### Immunoprecipitation

For immunoprecipitation, cells were lysed in buffer (20 mM Tris-HCl, pH 7.4, 150 mM NaCl, 1 mM EDTA, 1 mM EGTA, 1% Triton X-100) with protease (Roche Applied Science, Indianapolis, IN) and phosphatase (Pierce, Rockford, IL) inhibitor cocktails. Insoluble material was removed by centrifugation, and antibodies or control immunoglobulins were added to the lysates, followed by incubation at 4°C for 16 hours. Antibodies used for immunoprecipitation included monoclonal anti-Pim-1 (3 µg) and polyclonal anti-FLT3 (Santa Cruz Biotechnology, Santa Cruz, CA)**.** Immunocomplexes were collected using protein A- or protein G-sepharose beads, which were then washed extensively with lysis buffer three times at 4°C.

### Immunoblotting

Cells were lysed in buffer, as above, and immunoblotting was performed as described previously [30], [31]. Briefly, blots were incubated with primary antibodies, including 1∶400 dilution of monoclonal (Santa Cruz) and polyclonal (Cell Signaling Technology, Danvers, MA) anti-Pim-1, 1∶400 dilution of polyclonal anti-FLT3 (Santa Cruz), 1∶200 dilution of polyclonal anti-phospho-BAD at serine 112 (Cell Signaling Technology), 1∶1,000 dilution of polyclonal anti-BAD, monoclonal anti-phospho-c-Jun, monoclonal anti-c-jun, monoclonal phospho-Akt or monoclonal Akt (Cell Signaling Technology), 1∶2,000 dilution of monoclonal anti-glyceraldehyde 3-phosphate dehydrogenase (GAPDH) (Calbiochem, San Diego, CA), 1∶1,000 dilution of monoclonal anti-HSP90 (Santa Cruz), 1∶200 dilution of monoclonal anti-calnexin (Santa Cruz), 1∶100 dilution of monoclonal anti-phosphoserine (16B4, Calbiochem, San Diego, CA), 1∶1,000 dilution of monoclonal anti-phosphotyrosine (pY) (4G10, Millipore Co., Billerica, MA), 1∶200 dilution of monoclonal anti-ubiquitin (Santa Cruz), 1∶200 dilution of polyclonal anti-phospho-Y591 (Cell Signaling Technology) and 1∶500 dilution of anti-STAT5 and anti-phospho-STAT5 (Cell Signaling Technology), for 1 hour at room temperature or overnight at 4°C, followed by detection with horseradish peroxidase-conjugated secondary antibody. Densitometric scanning was performed with the Biospectrum AC imaging system (UVP, Upland, CA).

### Glutathione S-transferase (GST) pull-down assay

The GST pull-down assay was performed as described previously [30], [31]. Briefly, GST-tagged Pim-1 fusion protein or control GST (Cell Signaling Technology) was pulled down with glutathione beads for 1 hour at 4°C in 12 ml phosphate-buffered saline (PBS). The immobilized GST fusion protein or control GST was washed and resuspended in lysis buffer as above and incubated with an equal quantity of cell lysates (750 µg) for 1 hour at 4°C. The beads were washed four times with the lysis buffer and the protein complexes were then electrophoresed in a NuPage 4-12% Bis-Tris gel (Invitrogen, Carlsbad, CA), followed by immunoblotting with polyclonal anti-FLT3 antibody (Santa Cruz). FLT3 and GST-Pim-1 fusion proteins in the reactions were also measured, using polyclonal anti-FLT3 and anti-GST (Sigma-Aldrich), respectively.

### Pim-1 kinase inhibition

Pim-1 kinase was inhibited by incubating cells with the Pim-1-selective kinase inhibitor quercetagetin (Calbiochem) at 10 µM in 0.1% DMSO [36], [37], as we determined that quercetagetin does not also inhibit FLT3 (Figure S1). Quercetagetin inhibits Pim-1 with IC_50_’s of 0.34 µM and 5.5 µM in *in vitro* kinase and cell-based assays, respectively, while it inhibits Pim-2 kinase with a ten-fold higher IC_50_ of 3.45 µM [36]. Pim-1 kinase inhibition was confirmed by measuring phosphorylation of BAD at serine 112 (S112) by immunoblotting [14]. In confirmatory experiments, the Pim-1 kinase inhibitor AR00459339 (“AR339”), was used at 500 nM, the concentration at which it was previously demonstrated to inhibit Pim-1 and not to inhibit FLT3 [38]. AR339 was a generous gift from Array BioPharma, Boulder, CO.

### *in vitro* kinase assay

GST-tagged active human Pim-1 protein (Sigma-Aldrich) at 0.250 µg was incubated with 10 µM quercetagetin or DMSO control in 25 µl of 1x kinase buffer (Cell Signaling Technology) supplemented with 500 µM ATP (Cell Signaling Technology) at 30°C for 15 minutes. GST-tagged active FLT3 (571-993) protein (Sigma-Aldrich) at 0.5 µg was then added to the reaction mixtures and incubated at 30°C for 30 additional minutes. The reaction was then terminated by adding 9 µL of 4X LDS sample buffer (Invitrogen).

### Pim-1 gene knockdown

Cells were infected with lentivirus containing Pim-1 small hairpin RNA (shRNA) or non-target control according to the manufacturer's protocol (Sigma Life Science, St. Louis, MO). Briefly, 2.5×10^5^ cells were mixed with lentivirus in a 12-well plate, then cultured for 72 hours after infection. As a control, cells were also infected with equal amounts of plasmid expressing green fluorescent protein (GFP) (Sigma Life Science). Three days after infection with Pim-1 or control shRNA, Pim-1 expression was measured by immunoblotting, with GAPDH as a loading control, to confirm the expected effect or lack of effect on Pim-1 expression. Forty-eight hours after infection, cells were selected for puromycin resistance with 1 µg/ml puromycin for 7 days and then 0.5 µg/ml for 11 days, and stable clones were then isolated and analyzed. On immunoblotting, Pim-1 signal to- noise ratio was significantly low in the three cell lines studied. Pim-1 protein is degraded mainly in the proteasome, with a half-life of approximately 1.7 hours [39]. Hence, in order to unequivocally confirm knockdown of Pim-1, 2×10^6^ cells of Pim-1 shRNA as well as control shRNA-transduced cells were treated with the proteasome inhibitor MG132 (2 µM) for 3 hours. The MG132-treated samples were then immunoblotted for Pim-1 expression to confirm Pim-1 knockdown.

### Protein stability

Cells were cultured at a density of 1×10^6^ cells/ml in RPMI 1640 medium with 10% fetal bovine serum (FBS) and 100 µg/ml cycloheximide (CHX) (Sigma-Aldrich) to inhibit new protein synthesis, and residual cellular protein was measured at serial time points by immunoblotting.

### Inhibition of glycosylation

Mature or complex glycosylation was inhibited by incubating cells with 50 mM 2-deoxy-D-glucose (2-DG, Sigma-Aldrich) for 24 hours in the presence and absence of quercetagetin at 10 µM.

### Proteasome inhibition

To evaluate the role of proteasomal degradation in increased FLT3-ITD turnover in the presence of quercetagetin or AR339, cells were incubated with 10 µM quercetagetin or 500 nm AR339 in the presence and absence of the proteasome inhibitor carbobenzoxy-L-leucyl-L-leucyl-L-leucinal (MG-132; Calbiochem) at 1 µM.

### Ubiquitination assay

FLT3-ITD cells were treated with quercetagetin or DMSO control for 3 hours and then lysed in immunoprecipitation buffer as above with 1% SDS. Cell lysates were boiled for 10 minutes. Insoluble material was removed by centrifugation at 23°C and supernatant protein was quantified. To detect ubiquitinated FLT3, FLT3 was immunoprecipitated from the lysate, resolved in a NuPage 4–12% Bis-Tris gel, and immunoblotted with antibody to ubiquitin or FLT3.

### Plasmid constructs

FLT3-ITD cDNA in pMSCV-puro vector was a kind gift from Dr. Guido Marcucci, the Ohio State University, Columbus, Ohio. To generate the S935D and S935E FLT3-ITD mutants, the serine at position 935 of the FLT3-ITD vector construct was mutated to alanine (A), aspartic acid (D) or glutamic acid (E) using the Site-Directed Mutagenesis kit (Strategene, Santa Clara, CA) as per manufacturer’s instructions. The primers used for site-directed mutagenesis for S935A FLT3-ITD were sense: 5'-tttgactcaaggaaacggccagccttccctaatttg-3' and anti-sense: 5'-caaattagggaaggctggccgtttccttgagtcaaa-3', S935D FLT3-ITD were sense: 5'-tgactcaaggaaacggccagacttccctaatttgacttcg-3' and anti-sense: 5'-cgaagtcaaattagggaagtctggccgtttccttgagtca-3', and those for S935E FLT3-ITD were sense: 5'-tttgactcaaggaaacggccagccttccctaatttg-3' and anti-sense: 5'-caaattagggaaggctggccgtttccttgagtcaaa-3'.

### Selection of stable FLT3-ITD S935 point mutant clones

PT67 cells were transfected with S935 wild type FLT3-ITD and each of the S935 mutated FLT3-ITD plasmid constructs, S935A, S935D and S935E using FuGENE 6 transfection reagent (Roche Applied Science, Indianapolis, IN), according to manufacturer instructions. For each construct, stable clones were isolated using the selection antibiotic puromycin (2 µg/ml). The isolated stable clones (PT67 S935, PT67 S935A, PT67 S935D and PT67 S935E) were propagated in culture in DMEM medium supplemented with 10% FBS and 2 µg/ml puromycin.

### Drug-induced apoptosis

Cells were incubated with the FLT3 inhibitors midostaurin (LC Laboratories, Woburn, MA) or sorafenib (LC Laboratories) in the presence and absence of quercetagetin or quizartinib (Selleck Chemicals, Houston, TX) in the presence and absence of AR339. Apoptosis was detected by Annexin V/propidium iodide (PI) labeling (R&D Systems, Minneapolis, MN), measured by flow cytometry on a FACScan flow cytometer (Becton Dickinson, San Jose, CA) and analyzed with CellQuest Pro software, version 5.2.1, 2005 (BD Biosciences, San Jose, CA).

### Statistical analysis

Percentages of apoptotic cells following incubation under different conditions were compared by the Student t-test, two-tailed.

## Results

### Pim-1 directly interacts with FLT3

**Figure 1:**
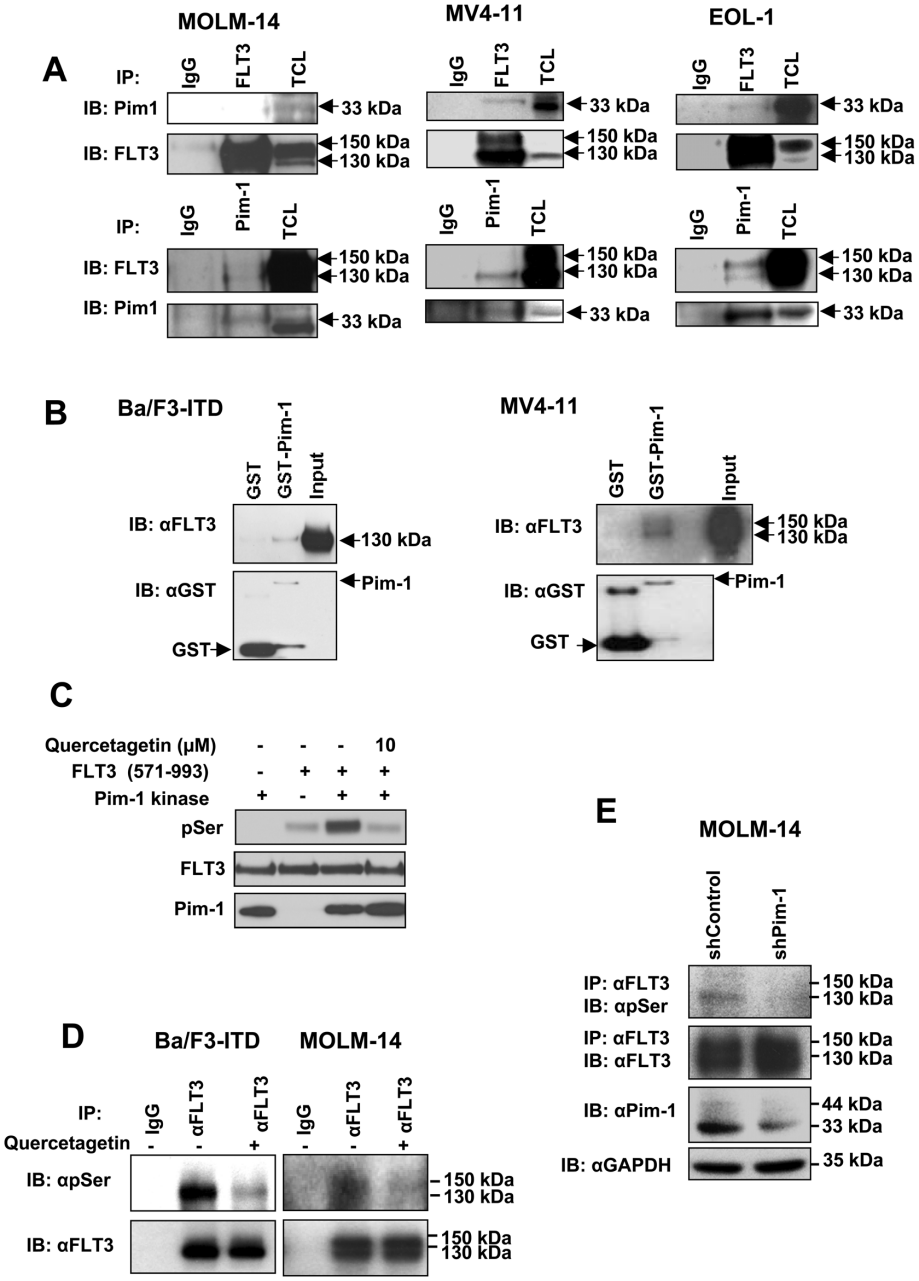
Pim-1 directly interacts with and serine phosphorylates FLT3. **A.** Pim-1 interacts with FLT3 in cells. Lysates of MV4-11 and MOLM-14 FLT3-ITD cells, and of EOL-1 FLT3-WT cells, all of which express Pim-1, were immunoprecipitated with anti-Pim-1 and immunoblotted with anti-FLT3, and, reciprocally, immunoprecipitated with anti-FLT3 and immunoblotted with anti-Pim-1. 10% of the total cell lysate (TCL) used for immunoprecipitation is shown as input control. Interaction between Pim-1 and FLT3 was detected in MV4-11 and MOLM-14, and also in EOL-1, indicating that Pim-1 interacts with both FLT3-ITD and FLT3-WT. **B.** Pim-1 interacts with FLT3 in vitro. Lysates from Ba/F3-ITD and MV4-11 FLT3-ITD cells were incubated with immobilized GST-tagged Pim-1 fusion protein or control GST, followed by immunoblotting with anti-FLT3 antibody. Total cell lysate is shown as input control. GST-Pim-1 recombinant protein, but not GST control, bound to FLT3-ITD, confirming direct binding of Pim-1 kinase to FLT3-ITD *in vitro*. **C.** Pim-1 interacts with and phosphorylates FLT3 on serine residues between amino acids 571 and 993. An in *vitro* kinase assay was performed with purified GST-FLT3 peptide (571-993) containing the Pim-1 consensus phosphorylation site at amino acid 935 (RKRPS) as the substrate and immunoprecipitates from MV4-11 cells with anti-Pim-1 antibody or IgG control on Sepharose A beads as the kinase, in the absence and presence of 10 µM quercetagetin, followed by immunoblotting with the antibodies indicated. Immunoprecipitates with IgG and with beads alone are shown as negative controls. Direct interaction between Pim-1 and FLT3 resulted in serine phosphorylation of FLT3, which w*a*s completely inhibited by 10 µM quercetagetin. **D.** Quercetagetin inhibits FLT3 serine phosphorylation by Pim-1. After treatment with 10 µM quercetagetin for three hours, FLT3-ITD was immunoprecipitated from BaF3-ITD and MOLM-14 cell lines and immunoblotted for phoshoserine and FLT3. Quercetagetin decreased FLT3 serine phosphorylation in both cell lines. **E.** shRNA knockdown of Pim-1 inhibits serine phosphorylation of FLT3. MOLM-14 cells were transiently transfected with control shRNA or Pim-1 shRNA for 72 hours. FLT3-ITD was immunoprecipitated and then immunoblotted for phosphoserine. The total cell lysates were also immunoblotted with anti-Pim-1 to confirm Pim-1 knockdown. GAPDH is shown as a loading control. Pim-1 knockdown decreased FLT3 serine phosphorylation, consistent with serine phosphorylation of FLT3 by Pim-1.

To test whether Pim-1 directly interacts with FLT3, co-immunoprecipitation and *in vitro* GST pull-down assays were performed. In the co-immunoprecipitation assays, lysates of MV4-11 and MOLM-14 FLT3-ITD cells, and of EOL-1 FLT3-WT cells, all of which express Pim-1, were immunoprecipitated with anti-Pim-1 and immunoblotted with anti-FLT3, and, reciprocally, immunoprecipitated with anti-FLT3 and immunoblotted with anti-Pim-1. Interaction between Pim-1 and FLT3 was detected in MV4-11 and MOLM-14, and also in EOL-1 (Figure 1A), indicating that Pim-1 interacts with both FLT3-ITD and FLT3-WT. In the *in vitro* GST pull-down assay, lysates from Ba/F3-ITD and MV4-11 cells, both with FLT3-ITD, were incubated with immobilized GST-tagged Pim-1 fusion protein or control GST, followed by immunoblotting with anti-FLT3 antibody. GST-Pim-1 recombinant protein, but not GST control, bound to FLT3-ITD (Figure 1B), confirming direct binding of Pim-1 kinase to FLT3-ITD *in vitro*.

### Pim-1 serine-phosphorylates FLT3

Next, to determine whether direct interaction between Pim-1 and FLT3 results in phosphorylation of FLT3 by Pim-1, an *in vitro* kinase assay was performed with purified GST-FLT3 peptide (571-993) containing the Pim-1 consensus phosphorylation site at amino acid 935 (RKRPS) as the substrate and immunoprecipitates from MV4-11 cells with anti-Pim-1 antibody or IgG control as the kinase. Direct interaction between Pim-1 and FLT3 resulted in serine phosphorylation of FLT3 (Figure 1C). In the same assay, presence of the Pim-1-selective kinase inhibitor quercetagetin [36], which does not inhibit FLT3 (Figure S1A), at 10 µM completely blocked serine phosphorylation of FLT3 by Pim-1 (Figure 1C). Because quercetagetin has also been reported to inhibit PI3 kinase and c-Jun N-terminal kinase (JNK) [40], we demonstrated lack of effect of quercetagetin at 10 µM on phosphorylation of the PI3 kinase target Akt or the JNK target c-jun (Figure S1B).

We then utilized quercetagetin to test the effect of Pim-1 inhibition on FLT3 serine phosphorylation in FLT3-ITD-expressing Ba/F3-ITD and MOLM-14 cells. Quercetagetin decreased FLT3 serine phosphorylation in both cell lines (Figure 1D). Next, to further confirm that Pim-1 phosphorylates FLT3 in FLT3-ITD cells, we performed shRNA knockdown of Pim-1 in MOLM-14 cells, and demonstrated that Pim-1 knockdown decreased FLT3 serine phosphorylation in MOLM-14 cells (Figure 1E), consistent with serine phosphorylation of FLT3 by Pim-1.

### Pim-1 inhibition decreases stability of 130 kDa FLT3

**Figure 2:**
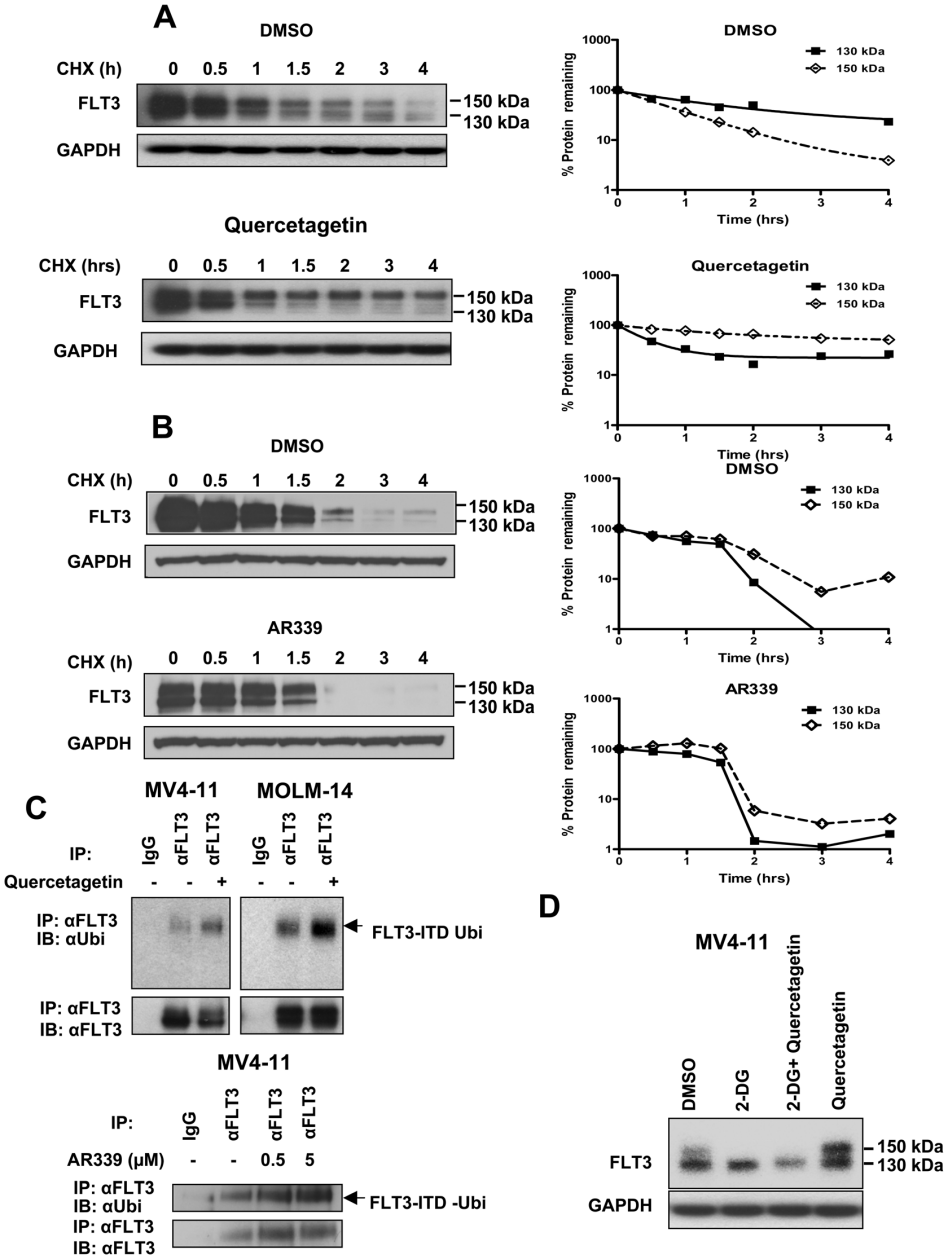
Pim-1 stabilizes 130 kDa FLT3-ITD A. Pim-1 inhibition with quercetagetin destabilizes 130 kDa and stabilizes 150 kDa FLT3. MV4-11 cells were treated with cycloheximide (CHX) in the presence or absence of 10 µM quercetagetin for the indicated time periods and immunoblotted for FLT3 and the loading control GAPDH. The expression ratio of each FLT3 isoform to GAPDH was quantitated by densitometric analysis, then normalized to “0” time point control and the normalized values are shown graphically as the percent of the FLT3 isoform remaining at various time points. **B.** Pim-1 inhibition with AR339 destabilizes 130 kDa and stabilizes 150 kDa FLT3. Cells were studied as in A, with 500 nM AR339. **C.** Pim-1 inhibition increases FLT3-ITD ubiquitination. MV4-11 and MOLM-14 cells were incubated with and without 10 µM quercetagetin for three hours and the protein lysates were immunoprecipitated with FLT3 antibody or control IgG and immunoblotted for ubiquitin. Total FLT3 in the protein lysates is also shown as control. A similar effect was seen in MV4-11 cells incubated with AR339 at a range of concentrations. **D.** Pim-1 inhibition enables glycosylation of 130 kDa FLT3, forming 150 kDa FLT3. MV4-11 cells were treated with 10 uM quercetagetin or DMSO control with and without the glycosylation inhibitor 2-deoxy-D-glucose (2-DG) for 24 hours, and immunoblotted with the indicated antibodies. Expression of 150 kDa FLT3 increased in the presence of quercetagetin, but this increase was abrogated by co-incubation with 2-DG, consistent with Pim-1 inhibition by quercetagetin enabling glycosylation of 130 kDa FLT3 to form 150 kDa FLT3.

Pim-1 is known to stabilize a number of its substrate proteins [22], [26]–[28]. Therefore we tested whether Pim-1 phosphorylation of FLT3-ITD enhances its stability. To accomplish this, we measured the half-life of FLT3-ITD following inhibition of new protein synthesis with cycloheximide in both the presence and absence of quercetagetin and of AR339. Pim-1 inhibition by quercetagetin or AR339 decreased the stability of 130 kDa FLT3 in MV4-11 cells, with half-life decreasing from an estimated 3 hours in DMSO control to an estimated 1 hour in the presence of quercetagetin (Figure 2A), and a similar decrease for AR339 (Figure 2A). In contrast, the apparent half-life of 150 kDa FLT3 increased from an estimated 1 hour in DMSO control to an estimated 3 hours in the presence of quercetagetin (Figure 2A), and also increased in the presence of AR339, although to a lesser degree. Simultaneous incubation with quercetagetin and the proteasome inhibitor MG-132 partially abrogated the effect of quercetagetin or AR339 on stability of 130 kDa FLT3 (Figure S2), indicating that Pim-1 protects 130 kDa FLT3 from proteasomal degradation. Moreover, quercetagetin treatment indeed increased ubiquitination of FLT3-ITD, detected by co-immunoprecipitation, in MV4-11 and MOLM-14 cells, and a similar effect of AR339 was seen in MV4-11 cells (Figure 2C). These data suggest that interaction with Pim-1 and serine phosphorylation by Pim-1 stabilize 130 kDa FLT3-ITD, preventing its complex glycosylation to form 150 kDa FLT3-ITD and protecting it from ubiquitination and proteasomal degradation. The observation that the apparent half-life of 150 kDa FLT3 increased in the presence of quercetagetin (Figure 2A) or AR339 (Figure 2A) additionally supports the hypothesis that Pim-1 inhibition might promote glycosylation of 130 kDa FLT3 to form 150 kDa FLT3. To test this hypothesis, we incubated MV4-11 cells with quercetagetin in the presence and absence of the glycosylation inhibitor 2-DG, and found that expression of 150 kDa FLT3 increased in the presence of quercetagetin, but that this increase was abrogated by co-incubation with 2-DG (Figure 2D), indeed consistent with Pim-1 inhibition by quercetagetin enabling glycosylation of 130 kDa FLT3 to form 150 kDa FLT3.

### Pim-1 knockdown decreases expression and stability of FLT3-ITD

**Figure 3:**
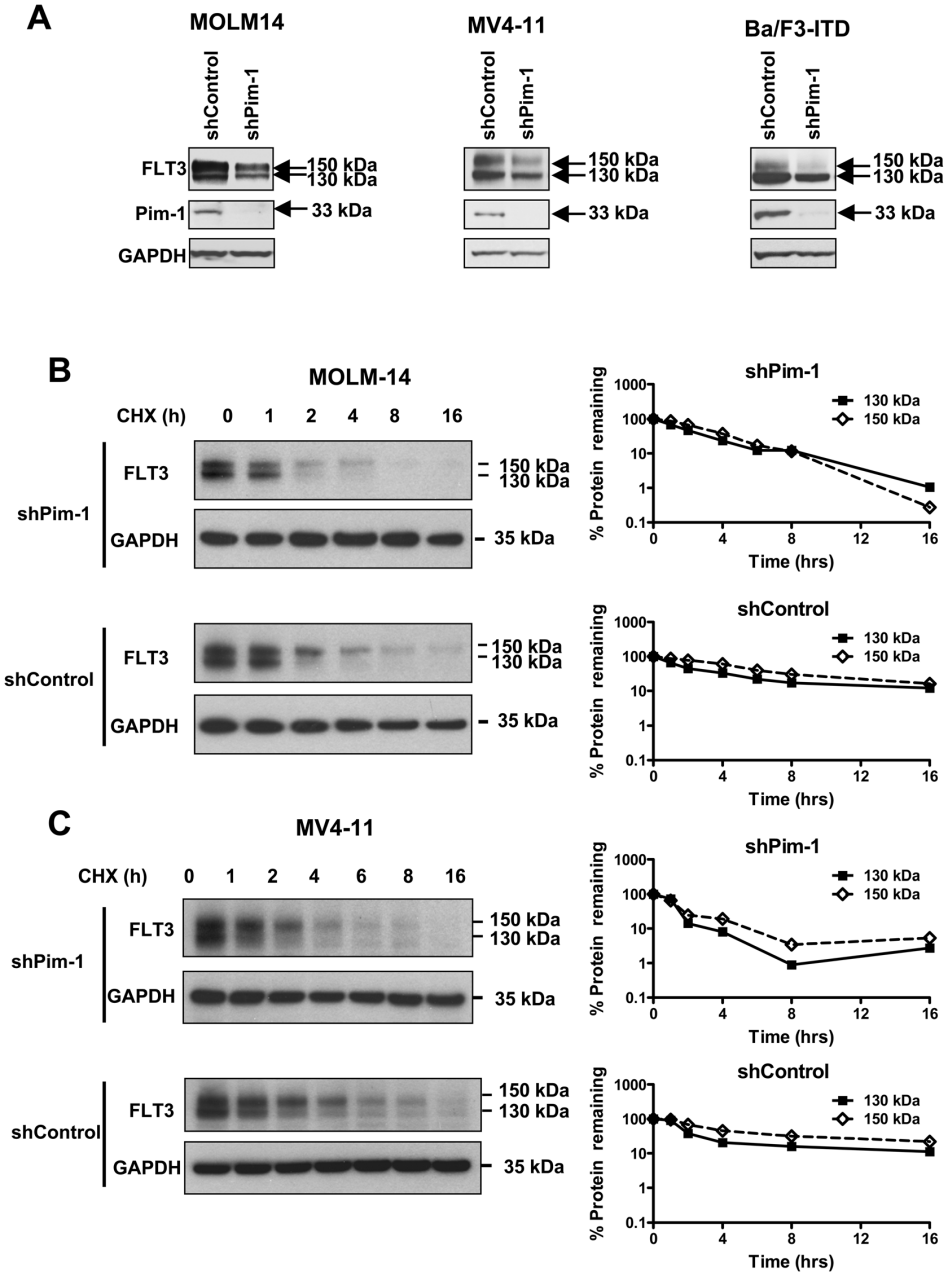
Pim-1 knockdown also destabilizes FLT3-ITD. **A.** Silencing Pim-1 with shRNA decreases FLT3-ITD expression. Pim-1 knockdown was performed as described in Materials and Methods, and expression of FLT3, Pim-1 and GAPDH control in Ba/F3-ITD, MOLM-14 and MV4-11 cells was measured by immunoblotting. Deceased expression of FLT3 was seen. (**B, C**). Pim-1 knockdown decreases half-life of 130 kDa FLT3. FLT3-ITD-expressing cells were treated with cycloheximide (CHX) combined with either 10 µM quercetagetin or DMSO for the times indicated. Expression of FLT3 and GAPDH control in MOLM-14 (**B**) and MV4-11 (**C**) cells was measured by immunoblotting. Decreased half-life of 130 kDa FLT3 was seen in both cell lines.

To confirm the specificity of the effect of Pim-1 kinase on stability of 130 kDa FLT3, FLT3 expression and stability were studied following Pim-1 knockdown in MV4-11, MOLM-14 and Ba/F3**-**ITD cells (Figure 3). Pim-1 knockdown decreased expression of FLT3 in Ba/F3-ITD**,** MOLM-14 and MV4-11 cells (Figure 3A). Of note Ba/F3-ITD, which are mouse cells transfected with human FLT3-ITD, differ from MV4-11, MOLM-14, in that they have a predominance of 130 kDa FLT3-ITD. Pim-1 knockdown also decreased the half-life of FLT3-ITD in MOLM-14 cells (Figure 3B) and in MV4-11 cells (Figure 3C).

### Effects of mutation of FLT3-ITD serine 935

**Figure 4:**
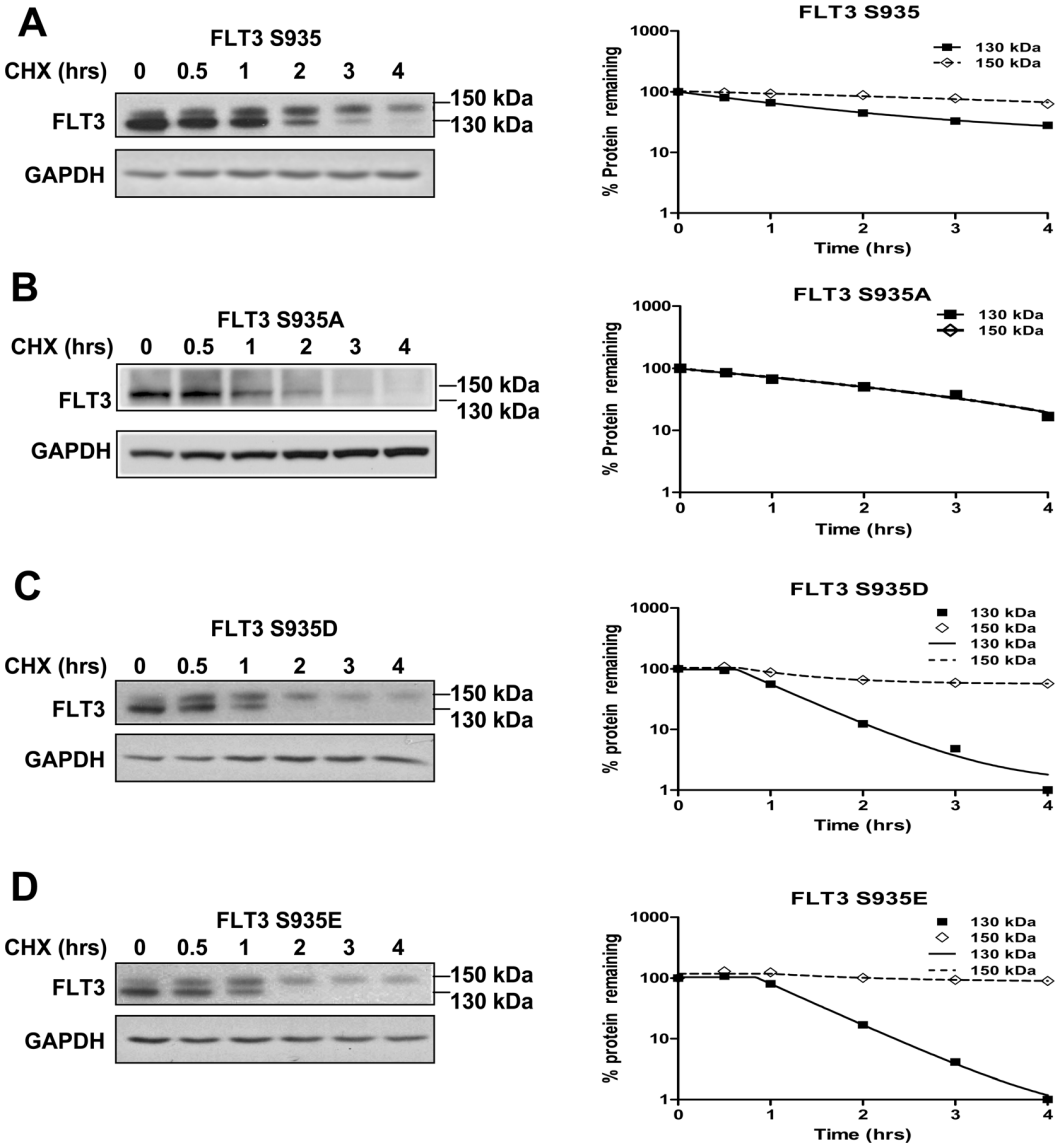
Mutation of serine 935 to alanine inhibits glycosylation of 130 kDa FLT3-ITD. Translation of FLT3-ITD in PT67 cells stably expressing the S935 (A), S935A (B) or phosphomimetic S935D (C) or S935E (D) FLT3-ITD constructs was blocked with cycloheximide and cells were collected at the indicated time points. Protein lysates were prepared and FLT3 and GAPDH expression was measured by immunoblotting following SDS-PAGE electrophoresis. A representative immunoblot is shown for each construct. The intensity of FLT3-ITD was quantified densitometrically and normalized in relation to that of GAPDH. Changes in intensity of GAPDH-normalized S935 (A), S935A (B), S935D (C) and S935E (D) 150 kDa and 130 kDa FLT3-ITD over time were then plotted graphically, in relation to 0 time point. The S935A mutant showed absence of 150 kDa FLT3, consistent with findings when glycosylation is inhibited by incubation with 2-deoxy-D-glucose, as shown in Figure 2D. The findings are consistent with the S935A mutation inhibiting glycosylation.

To further study the importance of serine 935, the Pim-1 consensus phosphorylation site, in stabilizing FLT3-ITD as a 130 kDa species, stability of FLT3-ITD S935, the S935A mutant and the phosphomimetic S935D and S935E mutants transfected into PT67 cells was studied by immunoblotting under reducing SDS-PAGE conditions following cycloheximide treatment (Figure 4, A–D). The non-phosphomimetic FLT3-ITD S935A mutant (Figure 4B) was expressed as a 130 kDa species (Figure 4A), and, notably, 150 kDa FLT3-ITD was not detected in S935A cells, consistent with absence of glycosylation, suggesting that serine 935 is an essential site for FLT3 complex glycosylation. Cross-talk between phosphorylation and glycosylation has been recently described and is being studied [41], [42].

### Pim-1 inhibition decreases FLT3-ITD association with calnexin and HSP90

**Figure 5:**
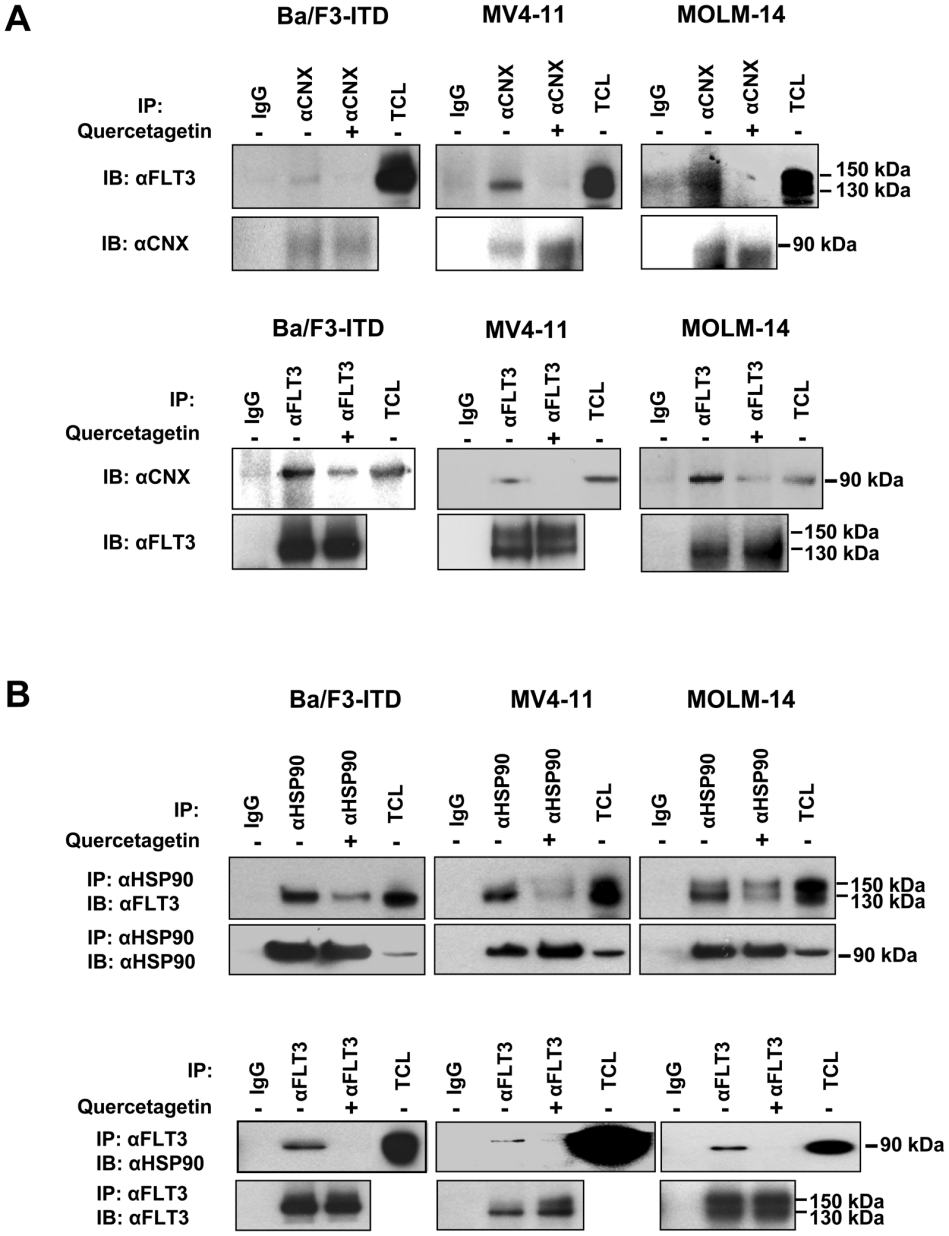
Pim-1 inhibition decreases FLT3-ITD binding to its chaperone proteins calnexin and HSP90. Ba/F3-ITD, MV4-11 and MOLM-14 FLT3-ITD cells were incubated with the Pim-1 inhibitor 10 µM quercetagetin or DMSO control for 3 hours, and protein-protein interaction was studied by immunoprecipitation (IP) followed by immunoblotting (IB) with the antibodies indicated. Treatment with quercetagetin disrupted FLT3-ITD binding to calnexin (**A**) and decreased its binding to HSP90 (**B**).

FLT3-ITD is known to be stabilized by interaction with the ER chaperone protein calnexin [8] and the cytosolic chaperone protein HSP90 [8, 10 and 11]. To test the effect of Pim-1 inhibition on FLT3-ITD interaction with calnexin and HSP90, Ba/F3-ITD, MV4-11 and MOLM-14 FLT3-ITD cells were incubated with the Pim-1 inhibitor quercetagetin (10 µM) or DMSO control for 3 hours, and protein-protein interaction was studied by immunoprecipitation followed by immunoblotting. Treatment of Ba/F3-ITD, MOLM-14 and MV4-11 FLT3-ITD cells with quercetagetin for 3 hours was found to disrupt FLT3-ITD binding to calnexin (Figure 5A) and to decrease its binding to HSP90 (Figure 5B). Thus Pim-1 phosphorylation appears to promote and be required for FLT3-ITD association with its chaperones calnexin and HSP90, likely contributing to stabilization of 130 kDa FLT3.

### Pim-1 inhibition decreases serine phosphorylation and activation of FLT3-ITD

**Figure 6:**
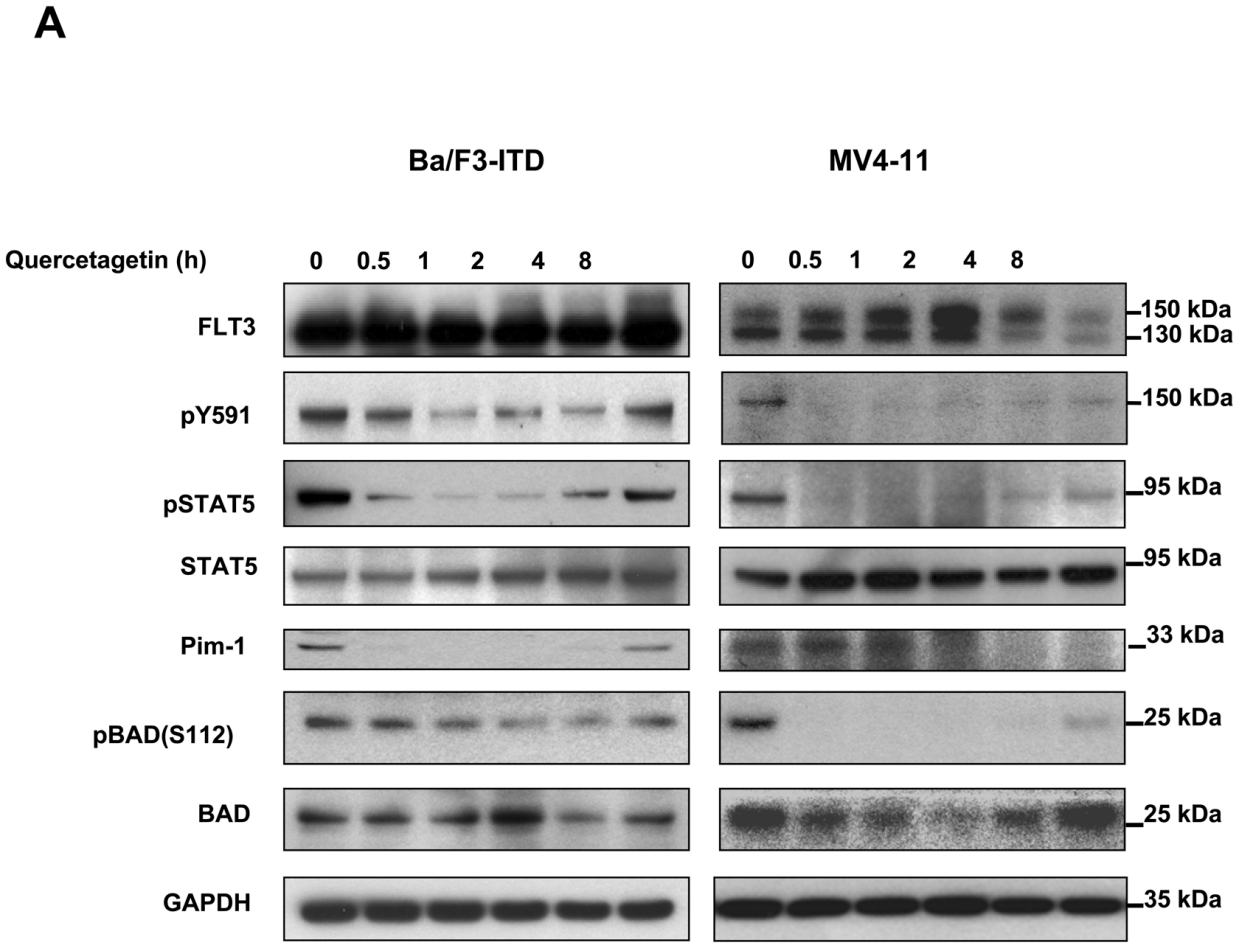
Pim-1 inhibition decreases FLT3 Y591 and STAT5 phosphorylation and Pim-1 expression in FLT3-ITD cells. Ba/F3-ITD and MV4-11 cells were treated with 10 µM quercetagetin or DMSO control for the times indicated, followed by immunoblotting (IB) with the antibodies indicated.

Because Pim-1 stabilizes 130 kDa FLT3-ITD and ER-retained 130 kDa FLT3-ITD mediates aberrant FLT3 signaling through STAT5 [9], [13], [14], which in turn increases Pim-1 expression, we hypothesized that Pim-1 inhibition would decrease STAT5 signaling and expression of Pim-1 itself. We therefore tested the effect of Pim-1 inhibition on the STAT5 pathway. Pim-1 inhibition decreased FLT3 phosphorylation at tyrosine 591 (pY591) (Figure 6), an essential site for binding of FLT3-ITD, but not FLT3-WT [43], to STAT5, and decreased STAT5 phosphorylation (Figure 6). Pim-1 inhibition also decreases expression of Pim-1 in FLT3-ITD cells and phosphorylation of the Pim-1 target BAD at S112 (Figure 6). The increase in pBAD, pSTAT5 and pFLT3 (pY591) levels observed from 4-8 hour incubation with quercetagetin suggest recovery of cells from quercetagetin inhibition after four hours of treatment, possibly due to short half-life of quercetagetin.

### Pim-1 and FLT3 inhibition produces a synergistic apoptotic effect in FLT3-ITD cells

**Figure 7:**
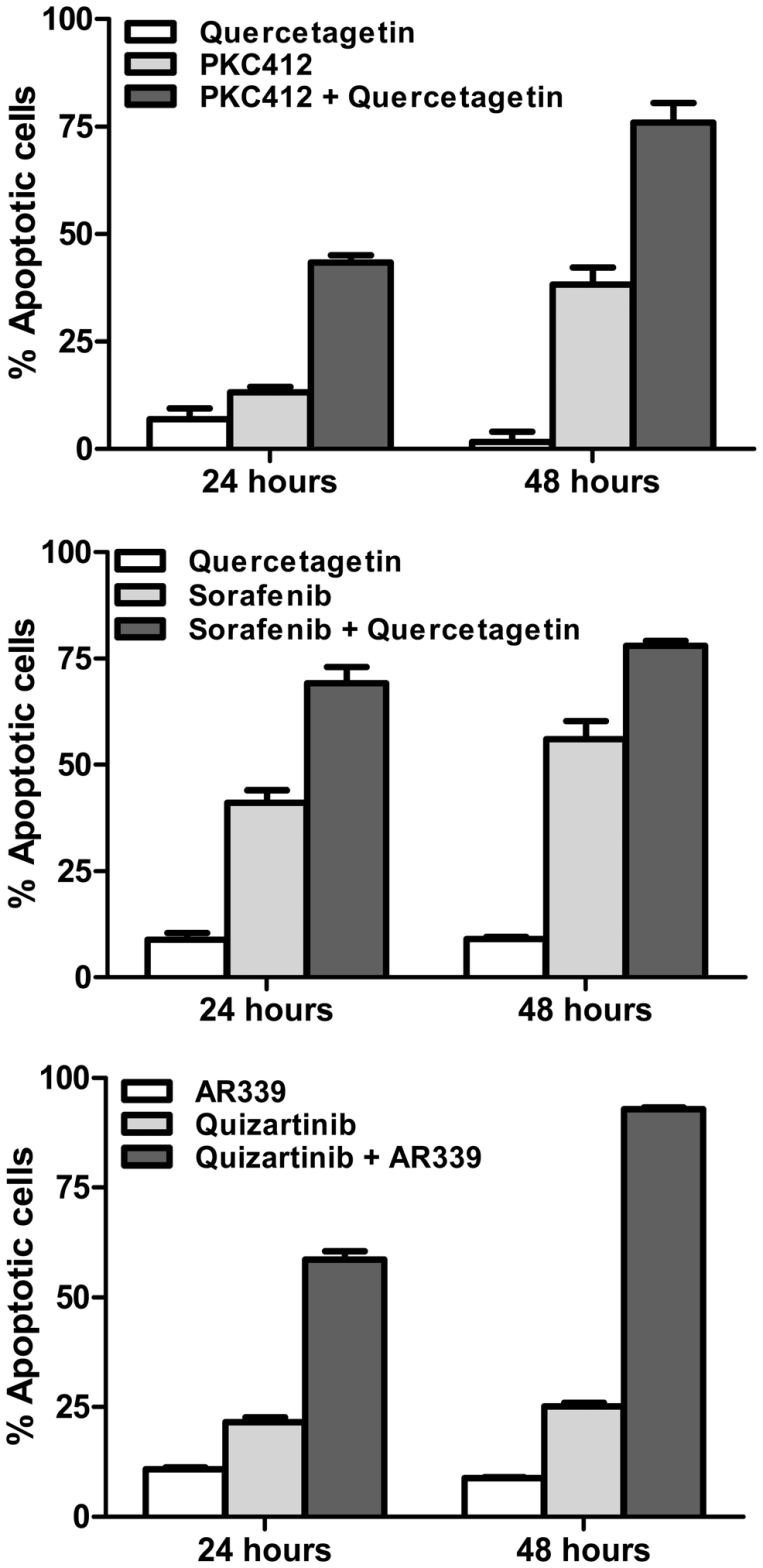
Pim-1 inhibition sensitizes FLT3-ITD cells to FLT3 inhibition. Ba/F3-ITD cells were treated with 100 nM PKC412 with and without 10 uM quercetagetin (top), and with 10 nM sorafenib with and without 10 uM quercetagetin (bottom) and also with quercetagetin alone for 24 and 48 hours, and apoptosis was measured by annexin V/propidium iodide staining, detected by flow cytometry and analyzed with CellQuest Pro software. Percentages of apoptotic cells were significantly greater following both 24-hour (p = 0.009) and 48-hour (p = 0.005) treatment with PKC412 and quercetagetin, compared with PKC412 alone, and following 24-hour (p = 0.03) and 48-hour (p = 0.05) treatment with sorafenib and quercetagetin, compared with sorafenib alone. The cells were also treated with the FLT3 inhibitor quizartinib at 1 nm with and without the Pim-1 inhibitor AR339 at 500 nM, demonstrating increased percentages of apoptotic cells at 24 and at 48 hours (p<0.001 for both).

Having demonstrated that, in addition to being a downstream effector of FLT3-ITD, Pim-1 stabilizes 130 kDa FLT3 and promotes STAT5 pathway signaling, we studied the combined effects of Pim-1 and FLT3 inhibition in FLT3-ITD cells. The Pim-1 inhibitor quercetagetin was found to synergize with the FLT3 inhibitors PKC412 and sorafenib in inducing apoptosis of FLT3-ITD cells (Figure 7). Percent apoptotic cells was significantly higher following 24-hour (p = 0.009) and 48-hour (p = 0.005) incubation with 100 nM PKC412 and quercetagetin, compared to 100 nM PKC412 alone, and following 24-hour (p = 0.03) and 48-hour (p = 0.05) incubation with 10 nM sorafenib and quercetagetin, compared to 10 nM sorafenib alone.

## Discussion

AML cells with FLT3-ITD are characterized by predominant expression of an underglycosylated, or high-mannose, 130 kDa FLT3 species that is retained in the ER and mediates aberrant signaling of STAT5, and the serine/threonine kinase Pim-1 is trancriptionally upregulated downstream of STAT5. We have demonstrated that Pim-1 in turn serine-phosphorylates and stabilizes 130 kDa FLT3 and thereby promotes aberrant signaling of STAT5, creating a positive feedback loop that sustains aberrant FLT3 signaling in AML cells with FLT3-ITD. We have additionally demonstrated that inhibiting both FLT3 and Pim-1 has enhanced efficacy in inducing apoptosis of FLT3-ITD cells, in relation to inhibiting FLT3 alone. Enhanced apoptosis resulting from direct inhibition of Pim-1, in addition to FLT3 inhibition, was recently described in another report [38], and has also been seen with knockdown of Pim-1 and Pim-2 [44]. Our data provide additional insight into regulation of this pathway and additional rationale for targeting Pim-1 in cells with FLT3-ITD.

While Pim-1 is known to phosphorylate a number of proteins that are key to regulation of cell proliferation, cell cycle and apoptosis, including BAD [16], [21], p21 [22], p27 [23], Cdc25A [24], Cdc25C [25], SOCS-1 [26], RUNX3 [27], and c-myc [28], to our knowledge it has not previously been shown to phosphorylate a mutant protein species and thereby promote aberrant signaling. We found that Pim-1 phosphorylates wild-type FLT3 as well as FLT3-ITD, but Pim-1 is expressed downstream of FLT3-ITD in cells with that mutation, thereby creating a positive feedback loop that promotes aberrant signaling in those cells.

The magnitude of both Pim-1 knockdown and Pim-1 inhibitor effects were variable among the three FLT3-ITD cell lines studied, likely reflecting different characteristics of these cell lines, as MV4-11 cells are homozygous for FLT3-ITD, while MOLM-14 is heterozygous [34], and Ba/F3-ITD is a human FLT3-ITD transfected murine cell line [35].

The mechanism by which Pim-1 serine phosphorylation stabilizes FLT3-ITD remains to be fully elucidated. FLT3-ITD undergoes ubiquitination and proteasomal degradation. The proto-oncogene c-Cbl, with ubiquitin ligase activity, promotes FLT3-ITD ubiquitination [12], and ubiquitinated FLT3-ITD undergoes degradation in the 26S proteasome [12]. We have demonstrated that Pim-1 phosphorylation protects FLT3-ITD from ubiquitination and proteasomal degradation. Moreover FLT3-ITD is known to be stabilized by association with its chaperones calnexin [8] and HSP90 [8, 10, and 11]. Similarly, the chaperones calreticulin and HSP90 have been shown to control expression of the human insulin receptor at its earliest maturation stages and to modulate its movement within the ER before either degradation or cell surface expression [45]. We have demonstrated that phosphorylation by Pim-1 enhances FLT3-ITD binding to HSP90, but whether Pim-1 directly forms a complex with HSP90-FLT3-ITD needs to be clarified. Of note, Pim-1 itself is an HSP90 client protein [39]. We also demonstrated that Pim-1-mediated phosphorylation enhances FLT3-ITD binding to its chaperone calnexin, which is likely important in mediating partial ER retention of 130 kDa FLT3-ITD [8]. We have previously shown that 33 kDa Pim-1, localizes to the cytosol in addition to the nucleus [30]. It is unclear whether Pim-1 also localizes to the ER. Since calnexin is a transmembrane chaperone, 33 kDa Pim-1 may interact with a transmembrane calnexin-FLT3-ITD complex adjacent to the ER membrane.

We sought to use mutational studies of the proposed FLT3-ITD Pim-1 phosphorylation site (S935) to confirm the decreased stability of the 130 kDa FLT3-ITD species observed on Pim-1 inhibition/knockdown. However mutating FLT3-ITD serine 935 to alanine did not mirror the effects of Pim-1 inhibition/knockdown on FLT3-ITD 130 kDa stability. Serines undergo competitive phosphorylation or O-linked glycosylation [41], [42], suggesting that mutation of serine at 935 most probably inhibits not only its phosphorylation by Pim-1, but also its complex glycosylation, thereby retaining FLT3-ITD as a 130 kDa species even in the absence of phosphorylation by Pim-1.

AML with FLT3-ITD is associated with adverse treatment outcome, and, in particular, short disease-free survival following chemotherapy. FLT3 inhibitors have been incorporated into treatment regimens for AML with FLT3-ITD, and have activity [3], but did not demonstrate improved treatment outcome in the single randomized clinical trial reported to date [4]. We have demonstrated that Pim-1 inhibition is synergistic with FLT3 inhibition in promoting apoptosis of AML cells with FLT3-ITD, as was also recently reported by another group [38]. Based on our data, Pim-1 inhibition should inhibit the FLT3-ITD signaling pathway upstream as well as downstream of FLT3-ITD. Of note, inhibition of the mTOR pathway at two levels has been shown to have greater efficacy in inhibiting the mTOR pathway, in relation to inhibition of this pathway at one level [46]. In the case of Pim-1 and FLT3, use of both inhibitors should not only inhibit the signaling pathway at two levels, but also interrupt a positive feedback loop.

Clinically applicable Pim-1 inhibitors are being developed and are entering clinical trials. Based on our data, combined administration of Pim-1 inhibitors and FLT3 inhibitors should enhance the clinical efficacy of inhibition of the FLT3 pathway in AML with FLT3-ITD. SGI-1776, the first Pim-1 inhibitor to have undergone phase I testing, is also a FLT3 inhibitor [47], [48]. Selective inhibition of Pim-1 but not FLT3 by quercetagetin was the basis for utilizing it as a Pim-1 inhibitor in this study, but quercetagetin is not clinically applicable. Other Pim-1 inhibitors in development inhibit Pim-1, but not FLT3; these include AR339 [38], also used here, as well as AZD1208 (unpublished data), the second Pim kinase inhibitor to enter clinical testing. Combining FLT3 specific and Pim-1-specific inhibitors may optimally inhibit aberrant signaling mediated by FLT3-ITD without undesirable off-target effects.

## Supporting Information

Figure S1 **A.** **The Pim-1 inhibitor quercetagetin does not inhibit FLT3.** In order to facilitate subsequent experiments it was essential to identify a Pim-1 inhibitor that did not also inhibit FLT3. Quercetagetin was known to inhibit Pim-1 kinase [31], but its effect on FLT3 had not been characterized. Hence FLT3-ITD- and Pim-1-expressing MV4-11 and MOLM-14 cells were incubated with and without quercetagetin at 10 µM for the indicated time periods, followed by immunoprecipitation of FLT3 and immunoblotting for both phosphotyrosine and total FLT3. Total FLT3 and GAPDH expression in the total cell lysates (TCL) is shown as input controls. Immunoprecipitataion with IgG is also shown as a negative control. Total tyrosine-phosphorylated FLT3, indicative of FLT3 tyrosine kinase activity, did not decrease in relation to total FLT3, demonstrating that quercetagetin does not inhibit FLT3 autophosphorylation. **B. Quercetagetin does not alter phosphorylation of the PDK1 target Akt or the JNK target c-jun.** MV4-11 cells treated with quercetagetin at 10 µM for the indicated time periods were immunoblotted for phospho-c-jun, total jun kinase and the loading control GAPDH, and phospo-Akt, total Akt and GAPDH. Phosphorylation of c-Jun, an indicator of JNK acitivity, and Akt, an indicator of PI3K activity, did not decrease with respect to total c-Jun or Akt expression, indicating absence of effect of quercetagetin on JNK and PI3K in the MV4-11 cell line.
(TIF)

Figure S2 **The proteasome inhibitor MG-132 overcomes destabilization of 130 kDa FLT3 by Pim-1 inhibition.** In the same experiments as in Figures 2, A and B, MV4-11 cells were treated with cycloheximide (CHX) and MG-132 in the presence or absence of 10 µM quercetagetin (**A**) and 500 nM AR339 (**B**) for the indicated time periods.
(TIF)
