# Supplementary figures and images for: Pim-1 Kinase Phosphorylates and Stabilizes 130 kDa FLT3 and Promotes Aberrant STAT5 Signaling in Acute Myeloid Leukemia with FLT3 Internal Tandem Duplication
Source: PLoS One. 2013 Sep 5;8(9):e74653. doi: 10.1371/journal.pone.0074653 (PMC3764066; doi:10.1371/journal.pone.0074653)

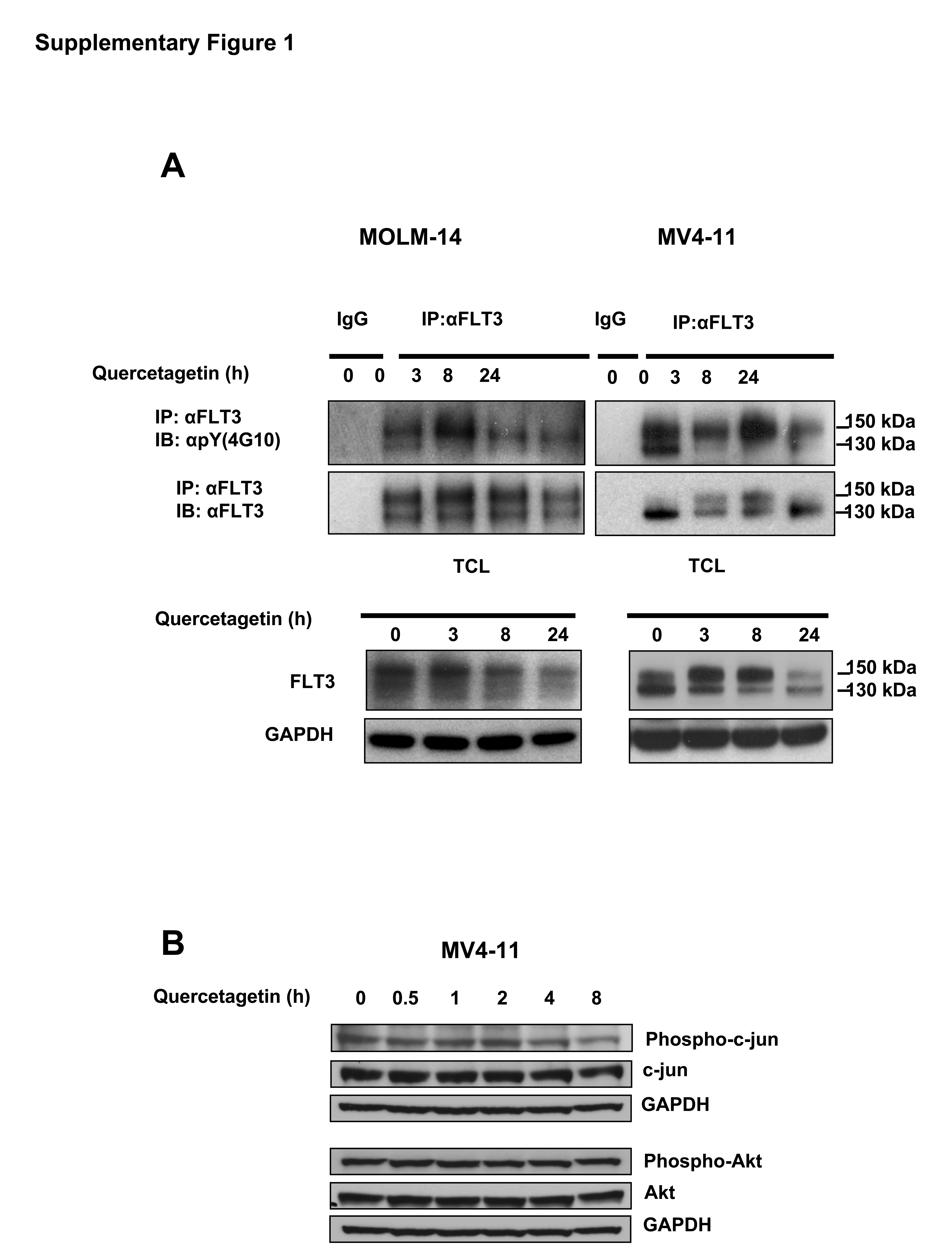

Supplement: Figure S1 — A. The Pim-1 inhibitor quercetagetin does not inhibit FLT3. In order to facilitate subsequent experiments it was essential to identify a Pim-1 inhibitor that did not also inhibit FLT3. Quercetagetin was known to inhibit Pim-1 kinase [31], but its effect on FLT3 had not been characterized. Hence FLT3-ITD- and Pim-1-expressing MV4-11 and MOLM-14 cells were incubated with and without quercetagetin at 10 µM for the indicated time periods, followed by immunoprecipitation of FLT3 and immunoblotting for both phosphotyrosine and total FLT3. Total FLT3 and GAPDH expression in the total cell lysates (TCL) is shown as input controls. Immunoprecipitataion with IgG is also shown as a negative control. Total tyrosine-phosphorylated FLT3, indicative of FLT3 tyrosine kinase activity, did not decrease in relation to total FLT3, demonstrating that quercetagetin does not inhibit FLT3 autophosphorylation. B. Quercetagetin does not alter phosphorylation of the PDK1 target Akt or the JNK target c-jun. MV4-11 cells treated with quercetagetin at 10 µM for the indicated time periods were immunoblotted for phospho-c-jun, total jun kinase and the loading control GAPDH, and phospo-Akt, total Akt and GAPDH. Phosphorylation of c-Jun, an indicator of JNK acitivity, and Akt, an indicator of PI3K activity, did not decrease with respect to total c-Jun or Akt expression, indicating absence of effect of quercetagetin on JNK and PI3K in the MV4-11 cell line. (TIF) [file pone.0074653.s001.tif]

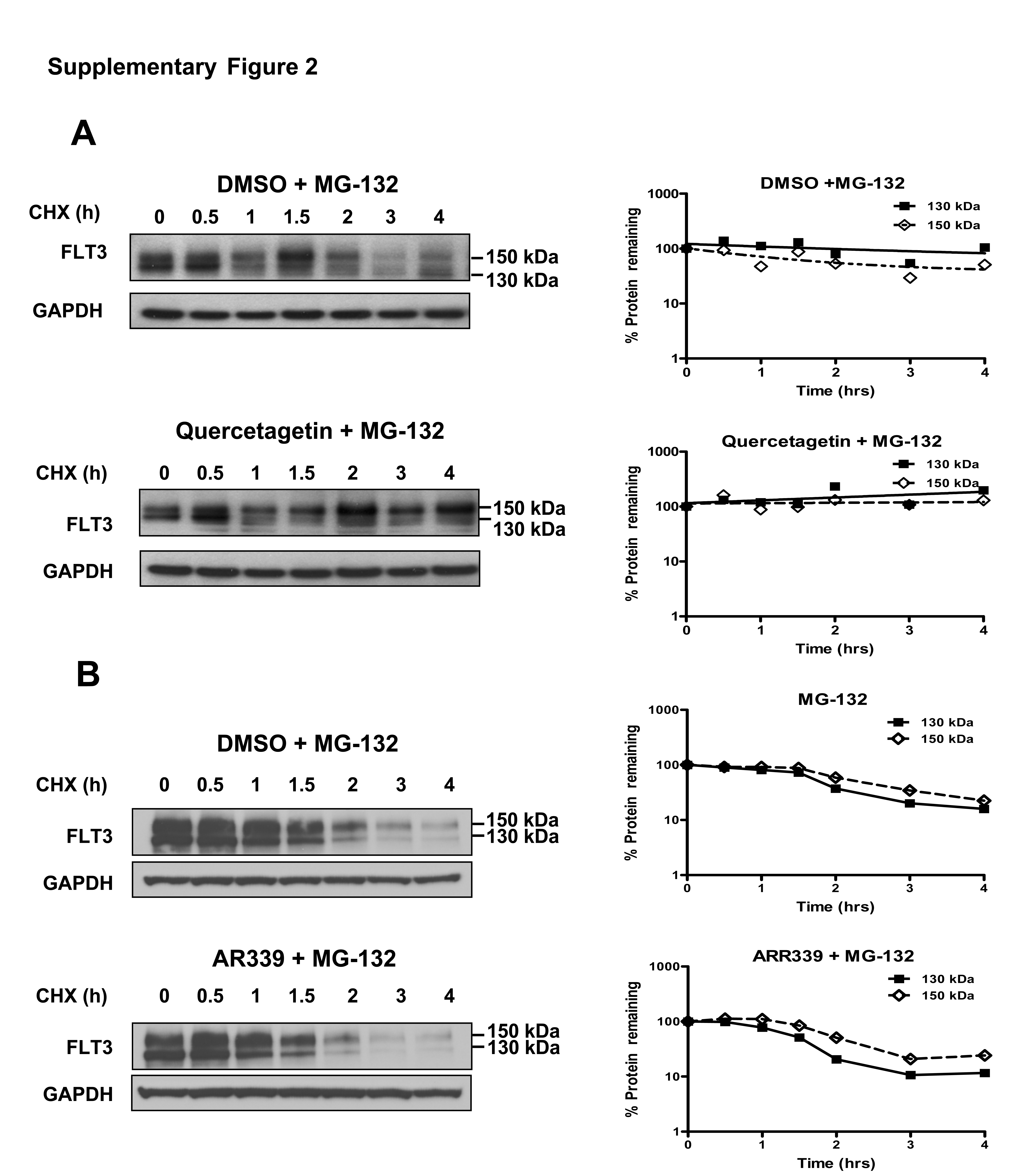

Supplement: Figure S2 — The proteasome inhibitor MG-132 overcomes destabilization of 130 kDa FLT3 by Pim-1 inhibition. In the same experiments as in Figures 2, A and B, MV4-11 cells were treated with cycloheximide (CHX) and MG-132 in the presence or absence of 10 µM quercetagetin (A) and 500 nM AR339 (B) for the indicated time periods. (TIF) [file pone.0074653.s002.tif]
